# Heat Shock Protein 27 is down-regulated in Ballooned Hepatocytes of Patients with Nonalcoholic Steatohepatitis (NASH)

**DOI:** 10.1038/srep22528

**Published:** 2016-03-03

**Authors:** Silvia Sookoian, Gustavo O. Castaño, Romina Scian, Julio San Martino, Carlos J. Pirola

**Affiliations:** 1 Department of Clinical and Molecular Hepatology, Institute of Medical Research A Lanari-IDIM, University of Buenos Aires–National Scientific and Technical Research Council (CONICET), Ciudad Autónoma de Buenos Aires, Argentina; 2 Liver Unit, Medicine and Surgery Department, Hospital Abel Zubizarreta, Ciudad Autónoma de Buenos Aires, Argentina; 3 Pathology Department, Hospital Diego Thompson, San Martin, Buenos Aires, Argentina; 4 Department of Molecular Genetics and Biology of Complex Diseases, Institute of Medical Research A Lanari-IDIM, University of Buenos Aires–National Scientific and Technical Research Council (CONICET), Ciudad Autónoma de Buenos Aires, Argentina

## Abstract

Ballooning degeneration (BD) of hepatocytes is a distinguishing histological feature associated with the progression of nonalcoholic fatty liver disease (NAFLD). Under the assumption that NAFLD severity is associated with metabolic-stress we explored the hypothesis that heat shock 27 kDa protein 1 (HSP27), a protein chaperone involved in stress resistance and cytoskeletal-remodeling, might be deregulated in ballooned hepatocytes. We observed that fasting plasma glucose (fpG) (*p* = 0.00002), total cholesterol (*p* = 0.02) and triglycerides (*p* = 0.01) levels, and female sex (*p* = 0.01) were significantly associated with the presence of BD. A logistic regression model showed that BD was independently associated with fpG (*p* = 0.002); OR per unit of glucose concentration 1.05, 95% confidence interval 1.02–1.09. Furthermore, BD was associated with a significant 2.24-fold decrease in the expression level of *HSP27*-mRNA in comparison with absence of ballooning, *p* = 0.002. Ballooned hepatocytes showed very low HSP27 immunoreactivity compared with hepatocyes without ballooning (*p* = 0.009); HSP27 immunoreactivity was inversely correlated with fpG levels (R: −0.49, *p* = 0.01). In conclusion, BD is associated with down-regulation of liver HSP27 gene and protein expression, suggesting that ballooned hepatocytes fail to ensure a robust physiological response to metabolic-induced stress.

Nonalcoholic fatty liver disease (NAFLD) is a worldwide prevalent chronic liver disease affecting not only adult but also pediatric populations^1^. From a clinical point of view, NAFLD has long been associated with all the components of the metabolic syndrome (MetSyn), including type 2 diabetes (T2D)^2^ and cardiovascular disease (CVD)^2^,^3^,^4^,^5^,^6^; in fact, NAFLD is regarded as the hepatic manifestation of the MetSyn^7^. From a histopathologic point of view, NAFLD refers to potentially progressive histological changes ranging from fatty liver alone (simple steatosis, NAFL) to nonalcoholic steatohepatitis (NASH), a disease stage characterized by liver cell injury, a mixed inflammatory lobular infiltrate, hepatocellular ballooning and mallory bodies (MBs), and variable fibrosis^8^. Notably, ballooning degeneration of hepatocytes is not only a key feature required for the diagnosis of NASH^9^ but seems to be a distinguishing histological finding associated with progressive NAFLD^10^. From a morphologic perspective, ballooning is characterized by swelling of the liver cells with rarefied cytoplasm. It has also been associated with injury of the cytoskeleton, involving an imbalance of the cytokeratin 8/18 ratio^11^,^12^. A remarkable study on the ultra structural exploration of ballooning degeneration showed that ballooned hepatocytes are enlarged, reaching more than 30 μm; in addition, ballooned cells accumulate both fat and osmiophilic droplets. Besides being deficient in cytokeratin 18, ballooned hepatocytes have degenerative changes that include areas of dilated endoplasmic reticulum^13^. Finally, an elegant study showed that hepatocytes undergoing ballooning degeneration generate hedgehog ligands, which act as pro-fibrogenic factors^14^. Although the phenotypic characterization of ballooned hepatocytes has been depicted thoroughly, the molecular events associated with this feature are still not fully understood.

Heat shock 27 kDa protein 1 (HSP27, also known as stress-responsive protein 27) is a protein chaperone with antioxidant properties that responds to cellular stress conditions rather than “heat shock”^15^. Under the assumption that NAFLD severity is associated with “metabolic-stress”^16^,^17^ we first sought to characterize the clinical and metabolic features associated with ballooning degeneration in a sample of patients who have biopsy**-**proven diagnosis of NAFLD. Further, we explored the hypothesis that ballooning degeneration is associated with deregulated gene and protein expression of HSP27.

## Results

### Ballooning degeneration is associated with metabolic stress

We first sought to characterize the clinical and metabolic stressors associated with ballooning degeneration in our population. Thus, we classified the explored variables into five groups: demographic and lifestyle factors; obesity and central obesity; glucose metabolism; CVD risk factors; and liver-related phenotypes. Then, we compared patients that developed hepatocellular ballooning with those that did not; ballooning was dichotomized to none versus mild or marked.

Of note, fasting plasma glucose (fpG) level was significantly associated with ballooning degeneration; the group of patients without hepatocellular ballooning showed significantly lower values of fpG levels than patients with marked ballooning (Table 1). Furthermore, female sex, total cholesterol and triglycerides levels, were significantly associated with the presence of ballooned hepatocytes (Table 1).

A logistic regression model was used to determine the independent associations of variables with the presence of ballooning degeneration; the analysis showed that ballooning was independently associated with fpG levels (*p* = 0.002), OR per unit of glucose concentration: 1.05, 95% CI: (1.02–1.09). Logistic regression analysis was performed considering sex, T2D status, plasma cholesterol levels, and liver enzymes (AST and AP) as independent variables and ballooning degeneration as the dependent variable (Table 2).

**Figure 1:**
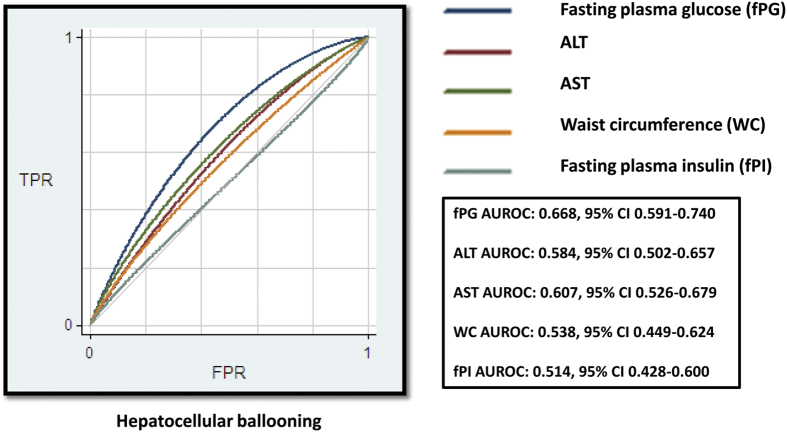
ROC curve analysis of the performance of plasma fasting glucose levels in predicting hepatocellular ballooning. The ROC analysis by main predictors associated with histologic disease progression, such as plasma fasting glucose and insulin levels, ALT, AST, and abdominal obesity evaluated as waist circumference was adjusted by body mass index. The figure indicates ROC curve analysis of the performance of plasma fasting glucose levels in predicting hepatocellular ballooning; our results show that glucose performed better than ALT, AST, waist circumference, and plasma fasting insulin levels in predicting ballooning degeneration (fPG vs. ALT *p* = 0.12, vs. AST *p* = 0.20, vs. WC *p* = 0.04, vs. fPI *p* = 0.05). fPG: fasting plasma glucose, fPI: fasting plasma insulin, TPR: true positive rate, FPR: false positive rate.

We observed that there was a trend of fpG (AUROC: 0.668, 95% CI: 0.591–0.740) toward to perform better than serum alanine and aspartate aminotransferases (ALT TGO and AST TGP) in predicting ballooning degeneration, while visceral obesity (waist circumference), and insulin levels were significantly (p < 0.02 and p < 0.01, respectively) better predictors of ballooning degeneration; Fig. 1 shows the results of the ROC analysis adjusted by covariates^18^.

### Liver HSP27 gene expression and protein levels are significantly down-regulated in the ballooned hepatocytes

The protein encoded by *HSP27* is induced by environmental stress and developmental changes; physiological stimuli including, oxidative stress and cytokines, increase the phosphorylation of HSP27 at different residues, which is essential for conferring resistance against actin-fragmentation^19^. Thereby, HSP27 is strongly involved in the modulation of structure and dynamics of components of the cytoskeleton^20^.

To contrast the hypothesis that ballooning degeneration could be associated with deregulated expression of HSP27, we explored changes in liver gene and protein expression in NAFLD patients with and without ballooning degeneration.

We found that ballooning degeneration was associated with a significant 2.24-fold decrease in the level of liver *HSP27*- mRNA (0.41 ± 0.21) in comparison with absence of ballooning (0.92 ± 0.37), *p* = 0.002 (Mann Whitney U Test).

**Figure 2:**
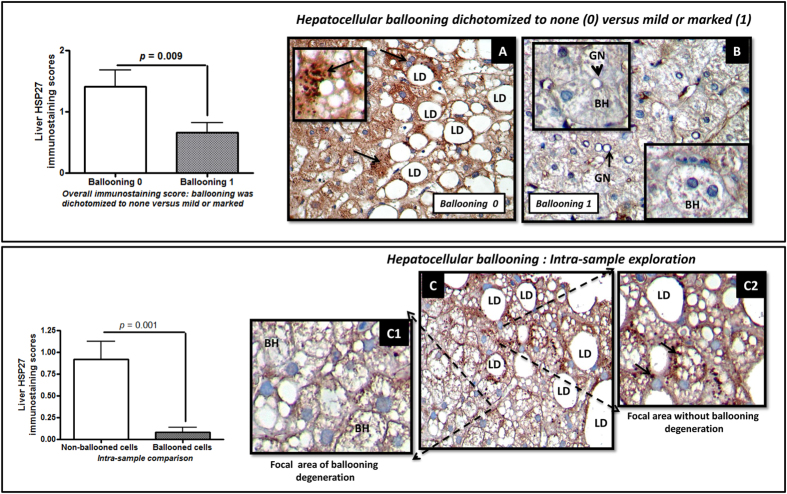
Liver expression of HSP27 is down-regulated in ballooned hepatocytes. Bars represent immunoreactivity scores of HSP27 staining; mean ± SD values. (**A**) Representative liver expression pattern of HSP27 in a NAFLD patient without ballooning degeneration; black arrows indicate HSP27 immunoreactivity in hepatocyte cytoplasm or surrounding the lipid droplets showing a granular pattern. (**B**) Representative liver expression pattern of HSP27 in a patient with NASH and ballooning degeneration. (**C**) Representative liver expression pattern of HSP27 in focal areas of marked ballooning degeneration (C1) or absence of ballooning (C2) in the same liver specimen of a patients with NAFLD. BH: ballooned hepatocytes (enlarged hepatocytes with pale to clear cytoplasm, some of them showing glycogenated nuclei GN); LD: lipid droplet. Protein expression was explored using immunohistochemistry; ballooning degeneration was dichotomized to: ballooning 0 (none) versus ballooning 1 (mild or marked). HSP27 immunoreactivity was examined using light microscopy of liver sections; counterstaining was performed with hematoxylin. Original magnification: 400X.

Furthermore, we observed that ballooning degeneration was associated with either no expression or very low HSP27 immunoreactivity compared with absence of ballooning (Fig. 2A,B).

In order to explain whether down-regulation of liver HSP27 is a finding associated with NASH -or the disease severity- rather than hepatocellular ballooning, we compared in the liver biopsy specimen of each patient, the HSP27 immunoreactivity in areas with ballooned hepatocytes versus those without. This strategy is supported by the fact that areas of ballooning degeneration and swelling of hepatocytes are often intermixed with areas of macro/micro vesicular steatosis with absence of ballooning^9^. Interestingly, this analysis showed an even more robust estimation of the significant down-regulation of HSP27 that was almost restricted to the areas of ballooning degeneration (Fig. 2C).

Finally, HSP27 immunostaining score was significantly and inversely correlated not only with the score of ballooning degeneration (R Spearman’s rank correlation: −0.72, *p* = 0.007) but serum liver enzymes levels (TGO: R: −0.89, *p* = 0.000075 and TGP: −0.85, *p* = 0.00036). In addition, HSP27 immunoreactivity was significantly and inversely correlated with type 2 diabetes (R: −0.59, *p* = 0.042) and fpG levels (R: −0.49, *p* = 0.01).

## Discussion

In this study, we aimed to contrast the hypothesis that ballooning degeneration is associated with an impaired capability of hepatocytes to maintain a protective stress-response; thereby, expression of HSP27 might be deregulated.

We first observed that abnormal glycemic control was the major metabolic determinant of ballooning degeneration; logistic regression analysis showed that ballooning degeneration was independently associated with glucose levels, which add about a 2–8% higher risk per unit of fasting plasma glucose concentration. We also observed that ballooning was associated with plasma total cholesterol and triglyceride levels, and female sex.

These findings are in agreement with a previous study that demonstrated that ballooning was associated with higher serum total cholesterol levels, abnormal glycemic control, and greater insulin resistance^21^. Nevertheless, we observed that high levels of glucose rather than insulin resistance are responsible for the putative toxic effect on the hepatocytes. It is known that constant exposure to hyperglycemia is associated with cellular dysfunction, which may become irreversible over time^22^. A similar line of reasoning might be transferred to the toxic effect of abnormal lipids levels^23^; from a morphologic perspective, evidence supports the concept that ballooning is the expression of accumulation of small droplets of fat (microsteatosis)^23^,^24^. Accordingly, at least in β cells and hepatocytes, glucose and lipotoxicity are interrelated rather than being separated phenomena, thus, it is biologically plausible that hepatocellular ballooning is the initial event leading to a progressive disease.

In addition, we found that ballooning degeneration was associated with a significant reduction of liver gene and protein expression levels of HSP27. Interestingly, we observed a close relationship between the levels of HSP27 in the liver and metabolic stressors (fasting plasma glucose) and surrogates of liver damage (liver enzymes). This relationship was inverse, indicating that the higher the level of metabolic stressors the lower the hepatic HSP27 expression. Of note, intra-specimen evaluation of liver HSP27 immunostaining scores showed significant differences between areas of ballooned hepatocytes vs. areas of absence of ballooning suggesting that the observed decrease in HSP27 protein expression was rather restricted to areas of ballooning degeneration than an association with the overall stratification of the disease severity.

While heat shock proteins were originally described in response to heat stress^25^, it is acknowledged that many stimuli can activate this response, including oxidative stress. After induction, HSP27 protects cells against cell death, including apoptosis and necrosis, and the aggregation of unfolded proteins^26^. Therefore, it is tempting to speculate that ballooned hepatocytes fail to ensure a robust physiological response to metabolic-induced stress. Unfortunately, lacks of an *in vitro* model that reproduce the morphological changes associated with ballooning degeneration preclude a demonstration of cause-effect. Thus, we cannot assure that down-regulation of HSP27 is the initial event that triggers subsequent molecular changes associated with ballooning degeneration or by the contrary, it is the consequence.

In conclusion, the results from our study suggest that ballooning degeneration is associated with decreased levels of liver HSP27 probably leading to an impaired ability of hepatocytes to deal with metabolic stressors, such as glucose. This scenario of decreased liver HSP27 might result in detrimental outcomes associated with NAFLD. Altogether, these observations support the concept that progressive NAFLD might be the consequence of the inability of hepatocytes to ensure a robust physiological stress-response^17^,^27^.

## Patients and Methods

### Study design and selection of patients

The investigations performed in this study were conducted in accordance with the guidelines of the 1975 Declaration of Helsinki. Written consent from individuals was obtained in accordance with the procedures approved by the Ethical Committee of our institution. The protocol was approved by the Comite de Etica Hospital Zubizarreta under protocol number: 104/HGAZ/09 and 89/100).

We included 256 patients with histopathologic evidence of NAFLD, either NAFL or NASH, on liver biopsies done within the study period. Secondary causes of steatosis, including alcohol abuse (≥30 g alcohol daily for men and ≥20 g for women), total parenteral nutrition, hepatitis B and hepatitis C virus infection, and the use of drugs known to precipitate steatosis were excluded. By using standard clinical and laboratory evaluation and liver biopsy features when applicable, autoimmune liver disease, metabolic liver disease, Wilson’s disease, and α-1-antitrypsin deficiency were likewise ruled out in all patients.

### Physical, anthropometric, and biochemical evaluation

Biological samples of patients included in this study we collected at study baseline before any intervention. All Health examinations included anthropometric measurements, a questionnaire on health-related behaviors, and biochemical determinations. For health-related behavior, the question about current smoking habit was asked as the number of cigarettes smoked per day. Regular physical activity was defined as all forms of activity, such as walking or cycling for everyday journeys; active play; work-related activity; active recreation, such as working out in a gym, dancing, or competitive sport; and the overall amount of activity was expressed in hours per week. There was no specific exercise intervention, and data regarding regular physical activity was surveyed at baseline by the time of liver biopsy.

The body mass index (BMI) was calculated as weight/squared height (kg/m^2^) and used as an index for relative weight. Additionally, the waist and hip circumferences were also assessed. Measurement of body fat content was performed using a bioelectrical impedance method at 50 kHz and 500 μA (OMRON Body Fat Analyser, model HBF-306, OMRON Healthcare, INC Illinois, USA). Abdominal wall thickness was measured using ultrasonography, and the minimum thickness of the subcutaneous fat was measured using longitudinal scanning with the use of a 7.5-MHz linear probe from the xiphoid process to the umbilicus along the linea alba.

Elevated blood pressure was defined as systolic arterial blood pressure (SABP) ≥ 130 mmHg and/or DABP ≥ 85 mmHg or receiving antihypertensive treatment. Determination of a 10-year risk of developing coronary heart disease outcomes (myocardial infarction and coronary death) was carried out using Framingham risk scoring^28^; the total cholesterol to high-density lipoprotein cholesterol (HDL) ratio was used additionally as a measure of cardiovascular (CV) risk.

### Biochemical determinations

Blood was drawn from 12-hour fasting subjects who had been in a supine resting position for at least 30 min. Laboratory evaluation of the subjects included include serum ALT and AST; gamma-glutamyl transferase (GGT); alkaline phosphatase (AP); glucose and insulin; total cholesterol; HDL and low-density lipoprotein (LDL) cholesterol; and plasma triglycerides. All biochemical determinations were measured using a Hitachi-912 Autoanalyzer (Roche, Diagnostic, Buenos Aires, Argentina) or Immulite 1000 (DPC, Buenos Aires, Argentina). Homeostasis Model Assessment (HOMA-IR) was used to evaluate an insulin resistance index and was calculated as follows: fasting serum insulin (μU/ml) × fasting plasma glucose (mmol/l)/22.5. Leukocyte count was measured automatically using a Sysmex XE 2100 equipment (Roche Diagnostics) (normal range: 6–10^9^/l). Serum C reactive protein (CRP) was measured in duplicate to evaluate low-grade inflammation by agglutination of the latex particles coated with antihuman CRP assay (CRP-Latex, BioSystems S.A., Barcelona, Spain) with a detection limit of 1.0 mg/L.

Anthropometric measurements and blood sampling were obtained from each patient at the time of liver biopsy and before any intervention.

Caspase-generated CK-18 fragment (CK-18)—a noninvasive quantification of hepatocellular apoptosis—concentration was measured by the one-step *in vitro* immunoassay M30-apoptosense ELISA kit (PEVIVA AB; DiaPharma, OH, USA) that recognizes selectively the caspase cleavage generated against the K18Asp396 neoepitope of CK-18.

### Liver biopsy and histopathologic evaluation

Liver biopsy was performed before any intervention with ultrasound guidance and a modified 1.4-mm-diameter Menghini needle (Hepafix, Braun, Germany) under local anesthesia on an outpatient basis. A portion of each liver biopsy specimen was fixed routinely in 40 g/l formaldehyde (pH 7.4) embedded in paraffin and then stained with hematoxylin and eosin, Masson trichrome, and silver impregnation for reticular fibers. All the biopsies were at least 3 cm in length and contained a minimum of eight portal tracts. A single experienced hepatopathologist, who was blinded to all clinical and laboratory data, read the liver biopsies. The degree of steatosis was assessed according to the system developed by Kleiner *et al*.^29^ based on the percentage of hepatocytes containing macrovesicular fat droplets: grade 0 = <5%; grade 1 = 5–33%; grade 2 = 34–66% and grade 3 = >66%. NASH was defined as steatosis plus mixed inflammatory cell infiltration, hepatocyte ballooning and necrosis, glycogen nuclei, Mallory’s hyaline, and any stage of fibrosis, including absent fibrosis. Intra-acinar (lobular) inflammation was defined according to Brunt^30^ as the presence of cellular components of inflammation (polymorphonuclear leukocytes, lymphocytes, and other mononuclear cells, eosinophils, and microgranulomas) located in sinusoidal spaces, surrounding Mallory’s hyaline, or in hepatocellular necrosis, and was graded 0–3. Intra-acinar (lobular) inflammation was defined as absent = no foci; 1 = <2 foci per 200 × field; 2 = 2 to 4 foci per 200 × field; 3 = >4 foci per 200 × field). Ballooning was scored as: 0 = none; 1 = rare or few; 2 = many. The severity of fibrosis was expressed on a four-point scale: 0 = none; 1 = perivenular and/or perisinusoidal fibrosis in zone 3; 2 = combined pericellular portal fibrosis; 3 = septal/bridging fibrosis; and 4 = cirrhosis. A NAS threshold of 5 was used for further comparisons with variables of interest^29^.

### Immunostaining for HSP27

Immunostaining for HSP27 was performed on a sub-sample of 20 liver specimens previously included in paraffin. Four-micrometer sections were mounted onto silane-coated glass slides to ensure section adhesion through subsequent staining procedures. Briefly, sections were deparaffinized, rehydrated, and washed in phosphate buffer solution (PBS), before being treated with 3% H_2_O_2_ in PBS for 20 min at room temperature to block endogenous peroxidase. Following microwave heat-induced epitope retrieval in 0.1 M citrate buffer at pH 6.0 for 20 min, the slides were incubated with a dilution of 1:100 of HSP27 antibody-C-terminal region ARP30177_T100 (Aviva Systems Biology, San Diego, CA, USA). Immunostaining was performed using the VECTASTAIN Elite ABC Kit (Vector Lab. CA, USA) detection system. Subsequently, slides were immersed in a 0.05% 3,3′-diaminobenzidine solution in 0.1 M Tris buffer, pH 7.2, containing 0.01% H_2_O_2_. After a brown color developed, slides were removed and the reaction was arrested by immersion in PBS. Hematoxylin was used as a counterstain. HSP27 immunostaining was semi-quantitatively evaluated in a blinded fashion regarding any of the histological and clinical characteristics of the patients by two independent observers.

The extent of staining was scored according to its amount and intensity, using a 4-point scoring system, as follows: 0 = no staining; 1 = positive staining in less than 20% of cells; 2 = 21–50% of positive cells, and 3 = positive staining in more than 50% of cells. The sections were observed by bright field microscopy, using a Axiostar plus (Carl Zeiss, Germany) microscope at a ×400 magnification.

### RNA preparation and real-time RT-PCR for quantitative assessment of mRNA expression

Total RNA was prepared from a sub-sample of 30 liver biopsy specimens in which nucleic acids were available to perform molecular studies; 14 patients with NAFLD without ballooning degeneration and 16 patients with NASH and ballooned hepatocytes were included in the molecular analysis.

RNA was purified from liver tissue using phenol extraction step method, with an additional DNAse digestion. For RT-PCR, 1 to 3 μg of total RNA was reverse-transcribed using random hexamers and Moloney Murine Leukemia Virus (MMLV) reverse transcriptase (Promega, Wisconsin, USA). Real-time PCR was performed for quantitative assessment of mRNA expression in a StepOne Plus Real-Time PCR Systems (Applied Biosystems, CA 94404, USA). All the real-time PCR reactions were run in triplicate.

The mRNA abundance of target genes was normalized to the amount of a housekeeping gene (*RPL19*) to carry out comparisons between the groups. The selection of the housekeeping gene was based on the exploration of the most stable reference gene for testing liver mRNA expression among other housekeeping genes tested before starting this experiment. The geNorm program^31^ was used to identify the appropriate reference control in our samples.

The mRNA levels were expressed as the ratio of the estimated amount of the target gene relative to the *RPL19* mRNA levels using fluorescence threshold cycle values (Ct) calculated for each sample, and the estimated efficiency of the PCR for each product was expressed as the average of all sample efficiency values obtained^32^. The specificity of amplification and absence of primer dimers were confirmed using the melting curve analysis at the end of each run. The primer sequences are shown in Table 3.

### Statistical analysis

Quantitative data were expressed as mean ± standard deviation (SD) unless otherwise indicated. Since a significant difference in the SD was observed between the groups in most of the variables and the distribution was significantly skewed in most cases, we chose to be conservative and assessed the differences between the groups using nonparametric Mann–Whitney *U* or Kruskal–Wallis tests. For the same reasons, correlation between two variables was done with the use of Spearman’s rank correlation test. A multivariate logistic regression analysis considering covariates as independent variables and histological features (ballooning degeneration, lobular inflammation and fibrosis, absent = 0, present = 1) as the dependent variable was performed separately on the whole group of patients. Significance was accepted at the *P* < 0.05 level. The CSS/Statistica program package version 6.0 (StatSoft, Tulsa, OK, USA) was used in these analyses unless indicated otherwise.

For plasma glucose levels, we constructed the receiver operating characteristic (ROC) curve and calculated the area under the ROC curve to evaluate the predictive power for liver histology using the free Web-based tool ROCCET (http://www.roccet.ca/ROCCET/) and the corresponding analysis for difference between AUROC was done by comproc subroutine as implemented in STATA 10.1 (Statacorp, College Station, TX, USA).

## Additional Information

**How to cite this article**: Sookoian, S. *et al*. Heat Shock Protein 27 is down-regulated in Ballooned Hepatocytes of Patients with Nonalcoholic Steatohepatitis (NASH). *Sci. Rep.* **6**, 22528; doi: 10.1038/srep22528 (2016).

## Figures and Tables

**Table 1:** Clinical and biochemical characteristics of the patients according to ballooning degeneration.

Variable	Ballooning (0 = none)	Ballooning (1–2)	P value
Number of subjects	143	113	
Demographic and lifestyle factors			
Male/Female, %#	59/51	30/70	0.01
Age, years	52.3 ± 11.7	50.7 ± 11.4	NS
Physical activity, h/week	1.2 ± 2.1	1.6 ± 6.0	NS
Alcohol consumption, g/day	17.5 ± 36	11.4 ± 26.6	NS
Obesity and central obesity			
BMI, kg/m^2^	31.7 ± 5.5	33.1 ± 6.2	NS
Waist circumference, cm	107 ± 12	108 ± 13	NS
Waist/hip ratio	0.97 ± 0.07	0.97 ± 0.07	NS
Body fat content, %	36 ± 8	38 ± 8	NS
Abdominal wall thickness, mm	49.5 ± 14	50.2 ± 14	NS
Glucose metabolism			
Fasting plasma glucose, mg/dl	101 ± 23	122 ± 43	0.00002
Fasting plasma insulin, mg/dl	14.6 ± 8.6	15.4 ± 10	NS
HOMA-IR index	3.7 ± 2.4	4.6 ± 4	NS
HbA1c, %	6.6 ± 1.6	7.8 ± 2.7	NS
Type 2 diabetes, %#	31	49	0.02
Cardiovascular risk factors			
SABP, mmHg	125 ± 16	129 ± 16	NS
DABP, mmHg	78 ± 11	80 ± 11	NS
Cardiovascular risk, %*	6.7 ± 9	6 ± 4	NS
Total cholesterol/HDL cholesterol ratio	3.7 ± 1.9	4 ± 1.9	NS
C reactive protein	5.7 ± 4	5.5 ± 4	NS
Leukocyte count, cells/mm^3^	7375 ± 2354	7769 ± 1890	NS
Total cholesterol, mg/dL	199.5 ± 41	216 ± 45	0.02
HDL-cholesterol, mg/dL	51.5 ± 19	51.4 ± 13	NS
LDL-cholesterol, mg/dL	121 ± 39	129 ± 40	NS
Triglycerides, mg/dL	152 ± 75	191 ± 117	0.01
Uric acid, mg/dL	5.2 ± 1.9	5.2 ± 1.8	NS
Liver phenotype			
ALT, U/L	58 ± 46	70 ± 49	0.06
AST, U/L	39 ± 20	48 ± 30	0.01
GGT, U/L	81 ± 87	73 ± 56	NS
AP, U/L	236 ± 107	193 ± 94	0.001
Total bilirubin, mg/dL	0.7 ± 0.3	0.7 ± 0.4	NS
CK-18&, UI/L	258 ± 270	307 ± 295	NS

Ballooning (0 = none; 1 = rare or few; 2 = many). Results are expressed as mean ± SD.; P value stands for statistical significance using the Mann–Whitney *U* test except where ^#^indicates the P value for a Pearson Chi square test. NS: non significant.

BMI: body mass index; SABP and DABP: systolic and diastolic arterial blood pressure, respectively; HOMA-IR: homeostatic model assessment-insulin resistance; ALT and AST: serum alanine and aspartate aminotransferase; GGT: gamma-glutamyl-transferase; AP: alkaline phosphatase. *Risk for developing coronary heart disease outcomes using Framingham risk scoring.

^&^Caspase-generated CK-18 fragment (CK-18): Caspase-generated CK-18 fragment (CK-18)—a noninvasive quantification of hepatocellular apoptosis—concentration was measured by the one-step *in vitro* immunoassay M30-apoptosense ELISA kit (PEVIVA AB; DiaPharma, OH, USA) that recognizes selectively the caspase cleavage generated against the K18Asp396 neoepitope of CK-18.

**Table 2:** Multiple logistic regression used to determine the independent associations of variables with the presence of ballooning degeneration.

	Sex	Diabetes	fPG	COL	AST	AP
p-level	0.12	0.42	0.002	0.10	0.75	0.009
Odds ratio (unit ch)	0.307	0.550	1.050	18.280	1.182	0.117
−95%CL	0.066	0.125	1.020	0.516	0.396	0.0225
+95%CL	1.425	2.413	1.090	646.513	3.531	0.607

fPG: Fasting plasma glucose, mg/dl; COL: Total cholesterol, mg/dL; AST: serum aspartate aminotransferase; AP: alkaline phosphatase.

**Table 3:** Primer sequences used for mRNA expression analysis.

Gene official name	HGNC Symbol Entrez Gene Ensembl number	Forward primer 5′→3′	Reverse primer 5′→3′
Ribosomal Protein L19	*RPL19 6143* ENSG00000108298	AAAACAAGCGGATTCTCATGGA	TGCGTGCTTCCTTGGTCTTAG
Heat Shock 27kDa Protein 1	*HSP27 3315* ENSG00000106211	ACGGTCAAGACCAAGGATGG	AGCGTGTATTTCCGCGTGA
